# The Implication of STEP in Synaptic Plasticity and Cognitive Impairments in Alzheimer’s Disease and Other Neurological Disorders

**DOI:** 10.3389/fcell.2021.680118

**Published:** 2021-06-14

**Authors:** Yacoubou Abdoul Razak Mahaman, Fang Huang, Kidane Siele Embaye, Xiaochuan Wang, Feiqi Zhu

**Affiliations:** ^1^Cognitive Impairment Ward of Neurology Department, The Third Affiliated Hospital, Shenzhen University, Shenzhen, China; ^2^Department of Pathophysiology, School of Basic Medicine, Key Laboratory of Education Ministry of China for Neurological Disorders, Tongji Medical College, Huazhong University of Science and Technology, Wuhan, China; ^3^Co-Innovation Center of Neuroregeneration, Nantong University, Nantong, China

**Keywords:** STEP, GluN2B, GluA2, Fyn, ERK1/2, synapses loss, cognitive impairment

## Abstract

STriatal-Enriched protein tyrosine Phosphatase (STEP) is a tyrosine phosphatase that has been implicated in Alzheimer’s disease (AD), the most common form of dementia, and many other neurological diseases. The protein level and activity of STEP have been found to be elevated in most of these disorders, and specifically in AD as a result of dysregulation of different pathways including PP2B/DARPP32/PP1, PKA as well as impairments of both proteasomal and lysosomal systems. The upregulation in STEP leads to increased binding to, and dephosphorylation of, its substrates which are mainly found to be synaptic plasticity and thus learning and memory related proteins. These proteins include kinases like Fyn, Pyk2, ERK1/2 and both NMDA and AMPA receptor subunits GluN2B and GluA2. The dephosphorylation of these molecules results in inactivation of these kinases and internalization of NMDA and AMPA receptor complexes leading to synapse loss and cognitive impairments. In this study, we aim to review STEP regulation and its implications in AD as well as other neurological disorders and then summarize data on targeting STEP as therapeutic strategy in these diseases.

## Introduction

Alzheimer’s disease (AD) is the most common form of dementia and is characterized by a gradual loss of short-term memory and a progressive decline of cognitive functions. It has been a major public health problem in modern society which will undoubtedly increase dramatically in the coming years, unless drugs that can prevent or cure the disease become available. According to the World Alzheimer Report, over 50 million people worldwide are currently living with dementia, and this number is estimated to reach 152 million by 2050 (Alzheimer’s Disease International, 2019). The two main histopathological hallmarks of AD are extracellular deposit of amyloid beta (Aβ) forming senile plaques, and intracellular hyperphosphorylated tau forming neurofibrillary tangles (Bennett et al., 2004). In addition to these, age-dependent synapse loss and the accompanying memory impairment are the next most common characteristics of AD patients as well as many AD models (Terry et al., 1991; Knobloch and Mansuy, 2008). Due to failure of many tau and Aβ based therapeutic strategies (Extance, 2010; Rinne et al., 2010), more drug development researches are now being shifted toward multitarget-directed ligands approaches like disease-modifying therapies (DMTs) which temporarily slow the worsening of dementia symptoms of those patients with AD and other dementias (Paoletti et al., 2013; Henley and Wilkinson, 2016). The 2020 Alzheimer’s disease drug development pipeline revealed that synaptic plasticity/neuroprotection agents in Phase 3 and Phase 2 clinical trials have reached up to 23.5% and 27.3% of DMT, respectively (Cummings et al., 2020), indicating that preventing and/or correcting alterations in synaptic functions in AD and other dementia patients might be a promising strategy in the management of these diseases.

Synapse has been regarded as a key target for different molecular assaults, like Aβ, that lead to the development and progression of AD, and synaptic dysfunction also correlates with the degree of cognitive decline in AD patients and transgenic AD mice (Terry et al., 1991). Glutamate receptors, including NMDA and AMPA receptors, play crucial roles in the mammalian central nervous system (CNS), where they involve in excitatory neuronal transmission and many other forms of synaptic plasticity (Paoletti et al., 2013; Henley and Wilkinson, 2016). Subunits that constitute NMDA receptors (NMDARs) include GluN1, GluN2A-D, GluN3A/B, while those that make up the AMPA receptors (AMPARs) are GluA1-4 (Henley and Wilkinson, 2016; Iacobucci and Popescu, 2017). NMDARs play central roles in brain development, synaptic plasticity, and learning and memory (Bliss and Collingridge, 1993; Aamodt and Constantine-Paton, 1999). Stimulating synaptic NMDARs activates pro-survival PI3K/AKt/CREB signaling pathways (Hardingham et al., 2002; Ivanov et al., 2006; Hardingham, 2009), which are involved in learning and memory formation. Interestingly, a reduced concentration of the GluN2B subunit of NMDAR and the postsynaptic density protein 95 (PSD-95), impaired long-term potentiation (LTP) and decreased NMDA and AMPA receptors’ currents in hippocampal CA1 region have also been reported in transgenic AD mice (Dewachter et al., 2009). It has been previously found that STriatal-Enriched protein tyrosine Phosphatase (STEP) is increased in AD, and opposes the development and strengthening of synapses via dephosphorylating and inactivating synaptic proteins including kinases such as Fyn, Pyk2, and ERK1/2 (Venkitaramani et al., 2009; Xu et al., 2012; Li et al., 2014). Besides, it can also lead to the dephosphorylation and internalization of synaptic receptor complexes like GluN2B/GluN1 and GluA2/GluA1 subunits of NMDA and AMPA receptors, respectively (Snyder et al., 2005; Zhang et al., 2008; Poddar et al., 2010; Wu et al., 2011).

STriatal-Enriched protein tyrosine Phosphatase is an intracellular phosphatase, enriched in the CNS except in the cerebellum, that is encoded by the PTPN5 gene, and is a member of a family of over a hundred protein tyrosine phosphatases (PTPs) (Lombroso et al., 1991, 1993), and it is one of the targets via which Aβ exerts its deleterious effects in AD. Elevated level of Aβ in AD is believed to be, at least in part, responsible for the activation of STEP via binding to and activation of the α7 nicotinic acetylcholine receptors (α7nAChRs) (Dineley et al., 2001; Stevens et al., 2003; Lacor et al., 2004). The activation of these receptors leads to increased calcium influx resulting in the activation of calcineurin, also known as protein phosphatase 2B (PP2B) (Stevens et al., 2003), and subsequent dephosphorylation and inactivation of DARPP-32, the inhibitor of protein phosphatase 1 (PP1). This process activates PP1, which then dephosphorylates STEP at the regulatory serine residue within the kinase-interacting motif (KIM) domain (Snyder et al., 2005), thereby activating STEP. Also, prolonged stimulation of NMDA receptors was found to dephosphorylate and activate STEP via the activation of the PP2B/PP1 pathway (Paul et al., 2003; Valjent et al., 2005). Dysregulations of STEP levels and activity have also been implicated in many neuropsychiatric disorders with cognitive dysfunctions including Parkinson’s disease (PD), Schizophrenia (SZ), Fragile-X syndrome (FXS), Huntington’s disease (HD) and others (Kurup et al., 2010, 2015; Zhang et al., 2010; Gladding et al., 2012; Chatterjee et al., 2018; Xu et al., 2018). The net result of this dysregulated function is alterations and mainly inactivation of many synaptic proteins including kinases and receptor complexes leading to learning and memory impairment, and cognitive deficits. In this study, we mainly summarized STEP isoforms, their activation and regulation via different posttranslational modifications, reviewed data on the implication of STEP in AD and other neuropsychiatric disorders, and finally highlighted the therapeutic strategies targeting STEP.

## Step Isoforms Expression, Posttranslational Modifications and Function

**FIGURE 1:**
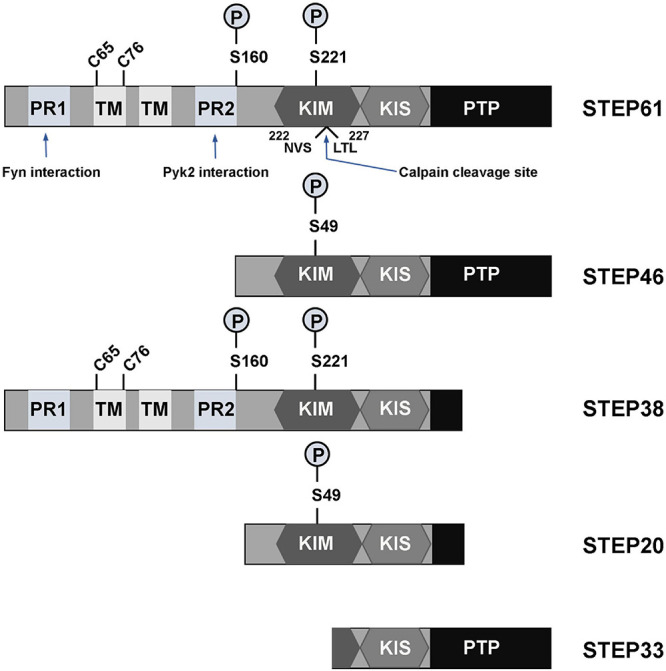
Isoforms and domains structure of STEP. The alternative splicing of *PTPN5* gene results in the production of four STEP isoforms (STEP61, STEP46, STEP38, and STEP20), while the fifth STEP isoform, STEP33, is the result of calpain cleavage of STEP61 at Ser224/Leu225 site. STEP61 and STEP46 are the fully active and major STEP proteins in the CNS. These two isoforms contain the KIM, KIS and PTP domains, which are respectively required for substrate interaction, specificity and phosphatase activity. STEP61 has two additional TM domains that serve for targeting it to the ER and PSD, as well as two PR (PR1 and PR2) regions that specifically provide binding with Fyn and Pyk2, respectively. The activity of STEP is inhibited via PKA phosphorylation at Ser221 and Ser49 within the KIM domain for STEP61 and STEP46, respectively. PKA can also phosphorylate STEP at Ser160 around the PR2 region, but its function is still unknown. The function of STEP can also be decreased by dimerization of STEP molecules via the C65 and C76 present within the TM domain. Together with STEP33 (the isoform that has disrupted binding domain), STEP38 and STEP20 are inactive variants since they have no PTP domain and thus lack phosphatase activity. Therefore, it is speculated that they may serve as negative regulators of STEP substrates by competitive binding. Adapted from Lombroso et al. (2016).

The family of STEP protein contains five isoforms that are presently known. Of these, four (STEP61, STEP46, STEP38, and STEP20) are the result of alternative splicing from the STEP gene (*PTPN5*), while the other one (STEP33) is the cleavage product of the protease calpain (Figure 1; Lombroso et al., 2016). Like other PTPs, the normal fully functional STEP contains a C-terminus catalytic signature consensus sequence [I/V]HCxAGxxR[S/T]G, and upstream KIM and kinase-specificity sequence (KIS) domains that allow the binding and specificity of STEP to its substrates, respectively (Bult et al., 1996; Pulido et al., 1998; Muñoz et al., 2003; Francis et al., 2014; Xu et al., 2015). The KIM domain is critical for binding, while KIS domain affects the binding, as evidenced by the fact that deleting both KIM and KIS domains decreased GluA2 binding to 7%, whereas deleting only the KIS domain decreased it to 45% (Won et al., 2019). In another study evaluating the effect of STEP on ERK1/2 phosphorylation, it was found that deletion of both KIM and KIS, KIM or KIS alone, or the C-terminal KIS resulted in a decreased k_cat_/K_m_ ratio by 50-60-fold, whereas deletion of the N-terminal KIS decreased the ratio by only 20-fold (Li et al., 2014). Moreover, mutations involving the conserved arginine residues or the hydrophobic motif around the KIM domain were found to decrease the k_cat_/K_m_ by 4-6-fold and 2.5-7-fold, respectively (Li et al., 2014). Also, deletion of the KIM domain decreased the ability of STEP interaction with both Fyn and Pyk2 (Nguyen et al., 2002; Xu et al., 2012). These further indicate that both KIM and KIS are required for efficient ERK, Fyn and Pyk2 dephosphorylation by STEP. The two fully active and most abundant forms of STEP are STEP61 and STEP46 which, are differentially expressed in the brain in terms of space and time (Boulanger et al., 1995; Sharma et al., 1995; Bult et al., 1997; Xu et al., 2015). STEP46 is a cytosolic protein, whereas STEP61 contains a unique 172-amino-acid domain at its N-terminus that targets it to the endoplasmic reticulum (ER) and both synaptic and extra-synaptic membranes (Boulanger et al., 1995; Oyama et al., 1995; Bult et al., 1996). STEP61 has two polyproline-rich regions that are necessary for Fyn (Nguyen et al., 2002) and Pyk2 (Xu et al., 2012) interactions (Figure 1). Both STEP46 and STEP61 isoforms are present in glial cells and neurons including excitatory and inhibitory neurons (Hasegawa et al., 2000; Lorber et al., 2004; Goebel-Goody et al., 2009), and are expressed in various regions of the brain including, but not limited to, the striatum, hippocampus and cortex (Boulanger et al., 1995; Lorber et al., 2004). While STEP46 is not expressed until day 6 postnatally, STEP61 is readily expressed in abundance at birth and throughout adulthood (Raghunathan et al., 1996; Okamura et al., 1997). The other two isoforms that result from alternative splicing (STEP38 and STEP20) do not have the PTP signature consensus sequence (Figure 1) and, therefore, are catalytically inactive (Sharma et al., 1995). But they both do contain KIM domain, indicating their ability to bind to target substrates and thus might protect from active STEP dephosphorylation. Several mechanisms including posttranslational modifications and others regulate the ability of STEP to bind and dephosphorylate its substrates. These processes include phosphorylation, ubiquitination, dimerization, proteolytic cleavage, and local translation.

**FIGURE 2:**
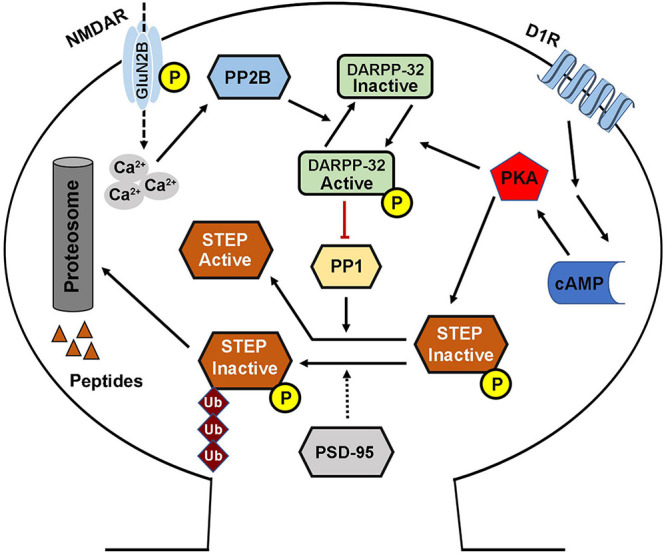
STriatal-enriched protein tyrosine phosphatase activity regulation. The phosphorylation (deactivation) of STEP is mediated by the D1R stimulation of cAMP synthesis that activates PKA. The activated PKA directly phosphorylates STEP in the KIM domain, and this inhibits the binding of STEP to its substrates. PKA can also indirectly mediate the phosphorylation of STEP via the phosphorylation and activation of DARPP-32, an inhibitor of PP1. This leads to the inhibition of PP1 activity, the STEP phosphatase, thereby increasing the phosphorylation of STEP. Phosphorylation of STEP downregulates its ability to bind and dephosphorylate its substrates. On the other hand, the dephosphorylation (activation) of STEP is mediated via NMDAR, and in other condition via α7nAChR, activation that induce intracellular calcium influx and activation of PP2B. The activated PP2B dephosphorylates and inactivates DARPP-32, thereby removing its inhibitory effect, leading to the activation of PP1 and thus increasing STEP dephosphorylation and activation. STEP level and thus activity could also be regulated via ubiquitination and proteasomal degradation which are mediated by synaptic activation of NMDARs, and could be enhanced by PSD-95.

The phosphorylation of STEP within the KIM domain decreases its ability to bind and dephosphorylate its substrates. This process is mainly regulated by two key enzymes including the cAMP dependent protein kinase A (PKA) and PP1 that are involved in its phosphorylation and dephosphorylation, respectively (Paul et al., 2000; Valjent et al., 2005). Directly, PKA phosphorylates STEP61 and STEP46 at regulatory Ser221 and Ser49 within their KIM domains, respectively (Paul et al., 2000), and thus sterically hindering STEP from binding to its substrates. Indirectly, PKA phosphorylates DARPP-32, a potent inhibitor of PP1, thereby maintaining STEP at its phosphorylated inactive state (Valjent et al., 2005). PKA can also phosphorylate STEP61 at Ser160 but its function is still unknown (Paul et al., 2000). The phosphorylation state of STEP could also be indirectly regulated by PP2B, which in the presence of increased intracellular calcium dephosphorylates and inactivates DARPP-32 thereby removing the inhibitory effect on PP1 which then dephosphorylates and activates STEP (Figure 2; Paul et al., 2003; Snyder et al., 2005; Valjent et al., 2005).

Moreover, the cellular level of STEP could be regulated by ubiquitin proteasome system (UPS) (Figure 2). This is evidenced by the finding that upon synaptic NMDAR activation, STEP is rapidly ubiquitinated and degraded (Xu et al., 2009), probably to decrease the dephosphorylation of STEP substrates and promote synaptic plasticity. In line with this hypothesis, it was found that following synaptic NMDAR activation, the phosphorylation of ERK1/2, a positive synaptic plasticity related protein, positively correlated with ubiquitination and degradation of STEP, leading to upregulation of dendritic spines’ size and density, and therefore, memory formation. However, the molecular mechanisms underlying ubiquitination of STEP are still unknown, but were speculated to be related to PEST sequences which were found at the amino terminal of STEP61 (Bult et al., 1996), as these sequences are often found in UPS degraded proteins (Spencer et al., 2004).

The activity of STEP could also be affected by two molecules of this protein itself coming together to form dimers. Under basal physiological conditions this dimerization process fundamentally occurs with STEP61, but not STEP46, via the formation of intermolecular disulfide bonds between the two cysteine residues (Cys65 and Cys76), that are present within the hydrophobic region of the amino terminus of STEP61 (Figure 1; Deb et al., 2011). However, oxidative stress can induce oligomerization of both STEP61 and STEP46 leading to a decrease in their phosphatase activity (Deb et al., 2011), possibly via involving additional sites other than Cys65 and Cys76. Interestingly, calpain can proteolytically cleave STEP61 between the Ser224 and Leu225 residues within the KIM domain (Figure 1), producing STEP33 that cannot associate with its substrates. It was found that STEP33 is produced after extra-synaptic NMDAR stimulation, and since STEP33 is inactive, this results in increased activation (phosphorylation) of the STEP61 substrate, p38, and initiation of cell death signaling pathways (Xu et al., 2009).

Local translation is another way to rapidly regulate STEP level in neuronal cells. Synaptic plasticity sometimes requires rapid translation of messages at distinct synapses via local translation. More importantly, this process is required in LTP as well as long-term depression (LTD) (Huber et al., 2000; Sutton and Schuman, 2006; Bramham and Wells, 2007; Costa-Mattioli et al., 2009). These plastic changes are possible due to the presence of mRNAs in a suppressed form along dendrites, until an appropriate synaptic stimulus ignites their translation (Bramham and Wells, 2007; Glock et al., 2017; Fonkeu et al., 2019). Interestingly, STEP was found to be locally translated as elaborated by the findings that STEP mRNA and protein were present in puncta along dendrites and near postsynaptic densities (PSDs), and its translation was upregulated within synaptosomes following (R, S)-3,5-dihydroxyphenylglycine (DHPG) activation of mGlu5 (Zhang et al., 2008). The dendritic local translation of STEP is believed to be regulated by the cytoplasmic polyadenylation element binding protein (Piqué et al., 2008) and fragile X mental retardation protein (FMRP) (Darnell et al., 2011; Goebel-Goody et al., 2012a; Chatterjee et al., 2018), that associate with, and repress STEP mRNA in dendrites until the arrival of appropriate stimuli, such as mGlu5 agonist activation. Moreover, there is evidence suggesting that STEP mRNAs, together with several other mRNAs, that are locally translated in response to synaptic activity, are shuttled by major vault protein to dendritic location (Paspalas et al., 2009).

## Step Activation and Regulation

The activity of the tyrosine phosphatase STEP is regulated by kinases and phosphatases as well as processes like dimerization. As mentioned above, the two main enzymes that regulate STEP activity are PKA and PP1. In normal conditions, STEP exists in a phosphorylated inactive state. This phosphorylation status is mainly due to the activation of PKA which can either directly or indirectly (Figure 2) maintain the phosphorylation of STEP and hence control its activity. Moreover, it has been demonstrated by previous studies that dopamine signaling can regulate STEP activity. In this model, the stimulation of dopamine D1 or blockage of D2 receptors was found to activate PKA which then phosphorylates and inactivates STEP, whereas the stimulation of D2 receptors had the opposite effects (Paul et al., 2000; Fitzpatrick and Lombroso, 2011). This is in support to the hypothesis that STEP is an intermediate bridge between the dopamine signaling and the glutamate signaling pathways, whereby dopamine regulates STEP activity and thus, tyrosine phosphorylation and surface expression of both NMDA and AMPA receptor complexes (Pelkey et al., 2002; Snyder et al., 2005; Zhang et al., 2008; Venkitaramani et al., 2011). In addition, the UPS regulates STEP level and thus its activity (Figure 2), via ubiquitination following synaptic NMDAR activation (Xu et al., 2009). Moreover, PSD-95 increases proteasomal degradation of STEP, and at the same time stabilizes NMDA receptors at the PSD favoring synaptic strengthening (Won et al., 2016). Interestingly, the expression of STEP in the PSD was increased in both PSD-95 knockdown neuronal cultures and PSD-95 KO mice (Won et al., 2016), indicating that PSD-95 is an important regulator of STEP.

On the other hand, stimulation of NMDARs was found to lead to a rapid but transient phosphorylation of ERK1/2, which has limited duration due to dephosphorylation and activation of STEP via the activation of the PP2B/DARPP-32/PP1 pathway (Paul et al., 2003; Valjent et al., 2005). The activated STEP can readily bind to its target proteins and lead to their dephosphorylation. It has been previously reported that there is a two- to three-fold increase in the level of STEP at the extra-synaptic sites as compared to synaptic sites (Goebel-Goody et al., 2009). This is supported by the findings that only extra-synaptic NMDAR expression and currents were increased upon STEP knockdown (Won et al., 2016, 2019). In a situation where glutamate levels increase at the synapse, there is subsequent activation of extra-synaptic NMDA receptors resulting in more calcium influx and activation of calpain which cleaves STEP61 into STEP33 that can no longer bind to and/or dephosphorylate its substrates (Xu et al., 2009; Lombroso et al., 2016). This decrease in the STEP activation promotes the activation of cell death signaling pathways via p38 (Xu et al., 2009). It is worth to note that recently conducted *in vitro* studies showed that STEP can be activated by a small molecule termed BI-0314 which binds to its phosphatase domain (Tautermann et al., 2019), however, further studies are needed before *in vivo* testing of this molecule.

## Step Substrates

STriatal-Enriched protein tyrosine Phosphatase acts via dephosphorylating its substrates, and the discovery of these substrates has elucidated the role played by STEP in neuronal signaling. Several proteins have been recognized as substrates of STEP, and many of them are related to learning and memory processes. These include subunits of both AMPA and NMDA receptors, kinases like ERK1/2, p38, Fyn, Pyk2 and other proteins such as PTPα and SPIN90 (Table 1).

**TABLE 1:** STriatal-enriched protein tyrosine phosphatase substrates dephosphorylation and the consequent effects.

**STEP substrates**	**Phosphorylation mechanism**	**Site**	**Consequences of STEP action**	**References**
GluN2B	Directly and indirectly via Fyn dephosphorylation	Tyr1472	Internalization of GluN1/GluN2B receptor complex and impaired synaptic plasticity	Nakazawa et al., 2001; Roche et al., 2001; Nguyen et al., 2002; Lavezzari et al., 2003
GluA2	Direct dephosphorylation	Tyr876	Internalization of GluA1/GluA2 receptor complex and impaired synaptic plasticity	Zhang et al., 2008; Won et al., 2016, 2019
ERK1/2	Direct dephosphorylation	Tyr204/187	Decreased ERK1/2 substrates (CREB, Elk1) phosphorylation, synaptic plasticity deficits	Paul et al., 2003; Valjent et al., 2005; Paul and Connor, 2010; Li et al., 2014
p38	Direct dephosphorylation	Tyr182	Inhibition of cell death pathways, enhanced cell survival	Xu et al., 2009; Poddar et al., 2010
Fyn	Directly and indirectly via Pyk2 dephosphorylation	Tyr420	Inhibition of Fyn substrates (GluN2B) phosphorylation	Nguyen et al., 2002; Venkitaramani et al., 2011
Pyk2	Direct dephosphorylation	Tyr402	Inhibition of Pyk2 substrates (Fyn) phosphorylation	Xu et al., 2012
PTPα	Direct dephosphorylation	Tyr789	Inhibition of Fyn dephosphorylation at inhibitory Try531 residue (Fyn inhibition)	Engen et al., 2008; Ingley, 2008; Xu et al., 2015
SPIN90	Direct dephosphorylation	Y85, Y161or Y227*	Activation of cofilin which depolymerizes actin leading to spine collapse, memory impairment	Cho et al., 2013a, b

*It is not confirmed which of these sites is/are STEP dephosphorylation residue (s).

The phosphorylation of GluN2B subunit of NMDARs is regulated by STEP via two different pathways, including direct dephosphorylation of Tyr1472 and the inactivation of Fyn, that phosphorylates GluN2B at the above-mentioned site (Nakazawa et al., 2001; Nguyen et al., 2002). Upon dephosphorylation by STEP, GluN2B binds to clathrin adaptor proteins which promote the internalization of GluN1/GluN2B receptor complex (Roche et al., 2001; Lavezzari et al., 2003). In concordance with this, it was observed that STEP KO mice showed an increased surface expression of GluN1/GluN2B receptor complex (Zhang et al., 2010; Venkitaramani et al., 2011). Moreover, it was found that increased amounts of STEP decreased NMDA receptors’ excitatory postsynaptic currents (EPSCs) and abolished LTP, while inhibition of STEP by anti-STEP antibody led to an enhanced EPSCs (Pelkey et al., 2002), and both genetic deletion (Olausson et al., 2012) and pharmacological inhibition (Saavedra et al., 2019) of STEP promoted LTP. Also, STEP ubiquitination and degradation following NMDAR stimulation ultimately permits the induction of LTP (Xu et al., 2009).

The other memory related receptor that is also regulated by STEP is AMPA receptor. It has been shown that trafficking of AMPA receptor occurred in LTD via its endocytosis from synaptic surface (Snyder et al., 2001; Hsieh et al., 2006). STEP was reported to regulate the Tyr dephosphorylation of the GluA2 subunit of AMPARs favoring the internalization of GluA1/GluA2 complex (Zhang et al., 2008; Won et al., 2019). Also, the surface expression of GluA1/GluA2-containing AMPA receptors was reported to be elevated in STEP KO mice (Zhang et al., 2008; Venkitaramani et al., 2011; Won et al., 2019). It was previously not clear whether STEP directly or indirectly induces the dephosphorylation and endocytosis of AMPARs complex since the Tyr876 of GluA2 can also be phosphorylated by SFKs (Hayashi and Huganir, 2004), which might also be dephosphorylated and inactivated by STEP. Additionally, GluA2 can directly interact with BRAG2 and activate Arf6, which then recruits adaptor protein-2 and clathrin to synaptic membranes (Krauss et al., 2003), to promote GluA2 endocytosis (Scholz et al., 2010). However, a recent study highlighted the mechanism of STEP regulation of AMPARs, whereby STEP binds to the C termini of GluA2 and GluA3, but not GluA1, to promote their tyrosine dephosphorylation (Won et al., 2019). Interestingly, in STEP overexpressing neuronal cultures, treatment with chloroquine (a lysosomal degradation blocker), but not MG-132 (a proteasomal degradation blocker), rescued GluA2/3 proteins and GluA2-PSD95 colocalization to control level (Won et al., 2019), indicating that STEP regulation of synaptic AMPARs is mediated by lysosomal degradation.

**FIGURE 3:**
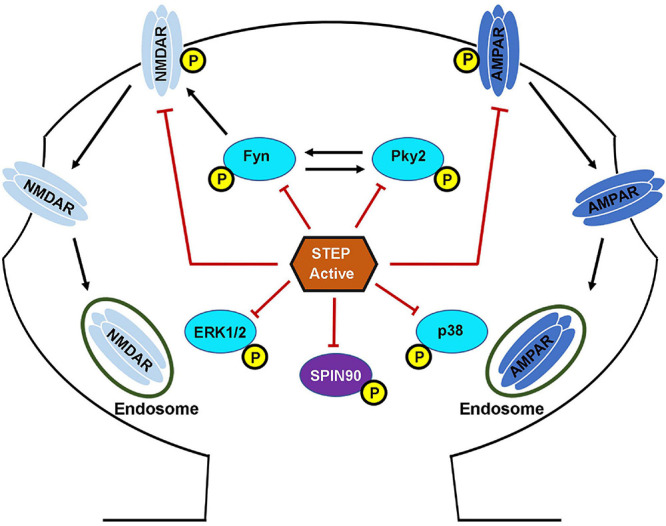
STriatal-enriched protein tyrosine phosphatase substrates. Following the NMDAR or α7nAChR stimulation mediated activation, STEP can then bind and dephosphorylate regulatory tyrosines within its substrates. The dephosphorylation of STEP substrates including GluN2B, GluA2\GluA3, Fyn, Pyk2, ERK1/2, p38 and SPIN90 leads to their inactivation. The dephosphorylation of GluN2B and GluA2\GluA3 subunits of NMDARs and AMPARs, respectively, results in internalization from the PSD. The dephosphorylation of Fyn, Pyk2, ERK1/2, and p38 leads to inhibition of their kinase activity and impairment of the downstream effects. The dephosphorylation of SPIN90 leads to its translocation to the dendritic shaft causing the release of its binding partner cofilin. STEP dephosphorylates GluN2B subunit of NMDARs by two mechanisms. First, STEP can directly dephosphorylate GluN2B at Tyr1472. Secondly, STEP can indirectly mediate the dephosphorylation of GluN2B via the inactivation of Fyn, the kinase that phosphorylates GluN2B at the same Tyr1472. The net result is endocytosis of GluN2B-containing NMDARs. STEP can also mediate the endocytosis of AMPARs via direct dephosphorylation of GluA2 and GluA3 subunits of these receptors.

Together, these findings indicate that fine-tuning of STEP activity is important for the regulation of proper levels of these glutamate receptors at synapses, since prolonged neuronal activity results in the upregulation of STEP that leads to the removal of NMDA and AMPA receptors from synaptic membranes, while prolonged neuronal inhibition has the opposite effect. Moreover, knocking down STEP in hippocampal slices increases AMPAR-mediated, but not NMDAR-mediated synaptic currents, while its overexpression reduced both synaptic expression and currents of AMPARs as well as NMDARs (Won et al., 2016, 2019). These facts together indicate that STEP preferentially regulates synaptic AMPA receptors, while on the other hand it regulates extra-synaptic NMDA receptors, suggesting a modulatory role of STEP in defining activity-dependent glutamate receptor localization. Thus, STEP is involved in the regulation of homeostatic synaptic plasticity (Jang et al., 2015) by regulating the surface expression of both NMDARs and AMPARs (Figure 3).

Other molecules that are involved in synaptic plasticity and memory, and have also been confirmed substrates of STEP are the two members of the MAPK family ERK1/2 and p38 (Muñoz et al., 2003; Paul et al., 2003; Kim et al., 2008; Paul and Connor, 2010). ERK1/2 is centrally implicated in synaptic plasticity and memory formation via several mechanisms including dendritic spines stabilization, local dendritic protein synthesis, nuclear transcription, and transmission of action potentials (Davis et al., 2000; Sweatt, 2004; Venkitaramani et al., 2009, 2011). The activity of ERK1/2 is dependent on its phosphorylation at the regulatory residues Thr202/185 and Tyr204/187 by MAPK kinases (MAPKK) such as MEK1/2 (Robinson and Cobb, 1997). To inactivate ERK1/2, the Tyr sites are dephosphorylated by STEP (Paul et al., 2003; Valjent et al., 2005; Paul and Connor, 2010; Li et al., 2014), and both KIM and KIS domains of STEP are required for ERK interaction (Li et al., 2014). Moreover, it was reported that ERK1/2 is necessary for the development of synaptic strengthening as well as the consolidation of fear memories in the amygdala, and STEP colocalizes with ERK1/2 in this brain area (Paul et al., 2007). Moreover, STEP KO mice showed a significant increase in the level of phospho-ERK1/2 and its downstream targets, CREB and Elk1, and improved hippocampal learning and memory (Venkitaramani et al., 2009, 2011). Also, UPS degradation of STEP led to the activation of ERK1/2, synaptic strengthening and neuronal survival pathways (Xu et al., 2009).

The p38 is another family member of MAPK and also a substrate of STEP (Poddar et al., 2010). However, in contrast to ERK1/2, p38 is implicated in cell death pathways and extra-synaptic NMDAR-mediated excitotoxicity (Ivanov et al., 2006; Semenova et al., 2007). As a phosphatase, STEP can dephosphorylate and inactivate p38 at the Tyr182 residue in the activation loop of p38 (Xu et al., 2009; Poddar et al., 2010). In this circumstance, STEP might play a protective role. However, the activation of extra-synaptic NMDA receptors leads to increased calcium and calpain activation, which in turn, cleaves STEP into an inactive STEP33 variant that is unable to bind to its substrates. This leads to increased phosphorylation and activation of p38 and thus activation of cell death signaling pathway (Xu et al., 2009). It should be noted that, synaptic NMDA receptor stimulation increases STEP activity which shortens the duration of p38 MAPK activation and favors neuronal survival, but extra-synaptic NMDARs stimulation causes significant degradation of active STEP via calpain-mediated proteolysis, leading to p38 MAPK activation (Poddar et al., 2010). This indicates that STEP serves as a modulator of NMDA receptor-mediated cell death by regulating p38 MAPK. On the other hand, both ERK1/2 and p38, can in turn, regulate STEP expression levels by modulating two phosphorylation sites (Ser59 and Thr72) within the KIS domain of STEP, dephosphorylation of which sites can trigger ubiquitination and thus degradation of STEP (Mukherjee et al., 2011).

Other substrates of STEP include Pyk2 and Fyn kinases. The two polyproline-rich (PR1 & PR2) regions of STEP (Figure 1) are implicated in substrate binding as well as specificity for Fyn (Nguyen et al., 2002) and Pyk2 (Xu et al., 2012), respectively. Upon binding, STEP can dephosphorylate the regulatory tyrosines in the activation loops of these kinases and inactivate them (Nguyen et al., 2002; Xu et al., 2012). Another identified substrate of STEP is PTPα, which is an activator of Fyn (Xu et al., 2015). STEP was reported to dephosphorylate PTPα at a Tyr789 site which, when phosphorylated, normally results in the translocation of PTPα to the lipid rafts to activate Fyn. PTPα dephosphorylates Fyn at an inhibitory Tyr531 residue in contrast to STEP which acts on the activation loop of Fyn at Tyr420 (Engen et al., 2008; Ingley, 2008). Thus, STEP can directly inactivate Fyn via Tyr420 dephosphorylation, or indirectly by dephosphorylating and blocking PTPα translocation to the membrane, thus maintaining the inhibitory Tyr531phosphorylation of Fyn.

**FIGURE 4:**
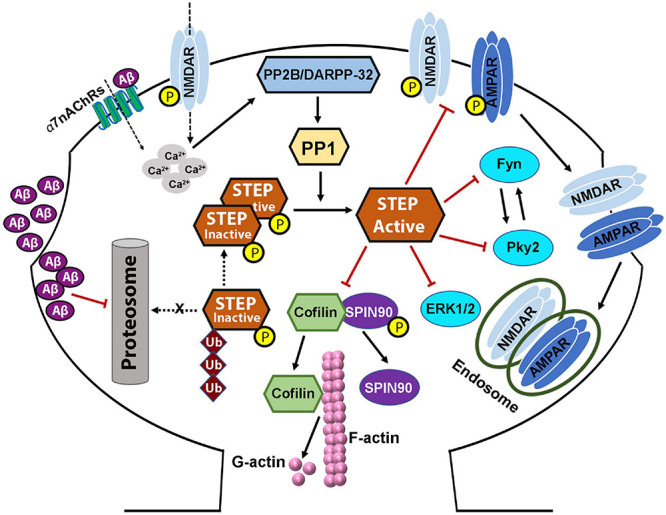
STriatal-enriched protein tyrosine phosphatase dysregulation in Alzheimer’s disease. Both protein level and activity of STEP are implicated in Alzheimer’s disease, and are thought to be the result of increased Aβ. Increased soluble Aβ levels precede the appearance of cognitive impairments. Aβ activation of α7nAChRs, together with glutamatergic NMDAR stimulation, increased calcium influx which then activates PP2B/DARPP-32/PP1 pathway leading to the dephosphorylation and activation of STEP. On the other hand, Aβ can also inhibit the proteasomal degradation of STEP leading to accumulation of STEP. These together lead to increased level of active STEP which aberrantly dephosphorylates its substrates. Dephosphorylation of SPIN90 by STEP also leads to the dissociation of SPIN90 from cofilin, leading to the activation of cofilin which then depolymerizes F-actin to G-actin. These events, together with the inactivation of other STEP substrates, and AMPA and NMDA receptors’ internalization, lead to dendritic spine and synapse loss resulting in the learning and memory and cognitive impairments seen in AD. Aβ-induced alterations in PKA/Akt/CREB pathway result in deficient BDNF/TrkB signaling, which in turn, contributes to the synapses loss and synaptic plasticity and cognitive deficits, via impairing the degradation of STEP.

SPIN90 is another substrate of STEP which in its phosphorylated form binds to and reduces the actin-depolymerizing activity of cofilin (Cho et al., 2013a). However, when SPIN90 is dephosphorylated by STEP (Figures 3, 4), it leads to cofilin activation and actin depolymerization, therefore, contributing to spine collapse (Cho et al., 2013b). Interestingly, it was found that loss of synaptic clustering with either Shank or PSD-95 following SPIN90 dephosphorylation by STEP affects both the size and density of dendritic spines (Cho et al., 2013a). The results from these studies indicate that SPIN90 dephosphorylation could be another way that STEP mediates Aβ-induced synaptic plasticity and memory impairments.

Recently, a study by Won et al. (2019) has identified 315 STEP interactors candidate proteins in WT mouse brain samples, including cytoskeletal-associated proteins and motor proteins like α-actinin, DBN1, myosin-10, MAP2, Arp2/3 complex; vesicle trafficking proteins like AP-2, Rab3a, SNX1, SNX4, NBEA; kinases and phosphatases like Fyn, PKA, PP2A, PP1; ion channels, receptors, and transporters like GluN2B, GluN1, GluA2, mGlu5; ATP synthase and ATPases like Na^+^/K^+^-transporting ATPase α-subunit; scaffolding proteins such as PSD-95, SynGAP, Kalirin, Shank; cell adhesion proteins like δ-catenin, NLGN-1; G protein-coupled receptor signaling proteins like Gα(o), Gα(q), Gβ-5; and ubiquitin enzyme proteins like Nedd4, RNF14, KCMF1. Among these, some proteins such as GluN2B and Fyn are already established STEP substrates, GluA2 recently confirmed, while many others will probably be confirmed in future studies.

## Step and Dendritic Spines

Loss of dendritic spines and decline of cognitive function are hallmarks of patients with AD and the loss of synapses correlated with cognitive deficits. Notably, in early stage of AD, studies revealed the existence of reduced dendritic spine density in the frontal cortex and hippocampal CA1 region of AD patients (DeKosky and Scheff, 1990; Scheff et al., 2006). Moreover, a decrease in the mushroom (memory) type spine density was reported in *in vivo* and *in vitro* Aβ toxicity (Popugaeva et al., 2015; Qu et al., 2017), cultures of hippocampal slice from AD transgenic mice (Tackenberg and Brandt, 2009; Penazzi et al., 2016), as well as in AD mouse models (Saito et al., 2014; Sun et al., 2014). Consistently, another study reported a shift from mushroom to stubby spines in cortical biopsies from AD patients (Androuin et al., 2018), indicating loss of memory-related spines. This loss of spines might be caused by Aβ peptide long before the appearance of clinical manifestations of the disease, that is, during the prodromal phase of AD, and was found to occur even before the disintegration of neuronal networks and consequent cognitive decline (Palop and Mucke, 2010; Kashyap et al., 2019). Several studies have revealed that transgenic AD mouse models as well as neurons exposed to Aβ show loss of spines (Lacor et al., 2004; Calabrese et al., 2007; Shankar et al., 2007; Wei et al., 2010; Spires-Jones and Hyman, 2014). Interestingly, it was recently shown that dendritic spine plasticity can provide cognitive resilience against dementia among AD patients (Boros et al., 2017).

It has been previously found that STEP is increased in AD and it was reported to oppose the development and strengthening of synapses via dephosphorylating and inactivating synaptic proteins including kinases like Fyn, Pyk2, and ERK1/2 (Venkitaramani et al., 2009; Xu et al., 2012; Li et al., 2014), as well as leading to the internalization of synaptic receptor complexes like GluN1/GluN2B and GluA1/GluA2 subunits of NMDA and AMPA receptors, respectively (Snyder et al., 2005; Zhang et al., 2008; Poddar et al., 2010; Wu et al., 2011; Won et al., 2019). Moreover, it was found that pharmacological inhibition as well as genetic depletion of STEP were able to ameliorate cognitive function and hippocampal memory in the 3×Tg-AD mouse model. In line with this, it was reported that STEP inhibition not only improved cognitive functions, but also increased synaptic connectivity in both cell cultures and 3×Tg-AD mouse model (Chatterjee et al., 2021), further highlighting the potential of STEP inhibitors as therapeutic agents. It has been reported that abundant ER are present in hippocampal dendritic spines and play an important role in synaptic plasticity (Holbro et al., 2009). Moreover, the ER in the spines exhibit highly dynamic changes that are largely dependent on NMDARs activity (Ng et al., 2014). Previous studies have also pointed out the involvement of STEP in the dynamics of dendritic spines, whereby dephosphorylation of SPIN90 by STEP led to cofilin activation and actin depolymerization to induce spine collapse, while elimination of STEP induced upregulation of dendritic ER-positive spines as well as dendritic spines’ ER growth (Cho et al., 2013b; Ng et al., 2014). Therefore, the upregulated ER changes observed upon STEP elimination might be, at least in part, due to the abrogation of the negative regulation of STEP on NMDAR.

Evidence from studies has indicated that LTD can induce removal of postsynaptic AMPA receptors and loss of spines (Snyder et al., 2001). Increased Aβ levels was shown to reduce pyramidal neuron spine density via LTD driven endocytosis of synaptic AMPA receptors, and removal of synaptic AMPA receptors was necessary and sufficient to produce dendritic spine loss and synaptic NMDA responses (Hsieh et al., 2006). Interestingly, it was recently reported that STEP can bind to and results in the dephosphorylation and decreased synaptic expression of GluA2 (Won et al., 2019), suggesting that STEP might mediate the Aβ-induced AMPA receptor internalization and synaptic impairment. Also, a 120 min Aβ treatment of cortical neurons resulted in increased STEP levels in these neurons with concomitant tyrosine dephosphorylation of STEP substrates, and a reduction in the protein levels of GluN2B receptors on membrane fractions (Kurup et al., 2010), while application of 1 μM of the STEP inhibitor TC-2153 was found to inhibit STEP activity in cortical cultures and restore the Tyr1472 phosphorylation of GluN2B receptor subunits (Xu et al., 2014).

Since studies have shown that Aβ leads to loss of spine in exposed neurons and that Aβ also increases STEP protein level and activity, it is interesting to investigate whether or not STEP is implicated in spine loss. Recently, a study reported that treatment of 18-day-old cortical neurons with conditioned media from mutant CHO cells containing Aβ showed significant decrease in dendritic complexity (dendritic junctions or nodes and Ends), as analyzed by Sholl analysis, compared to control neurons (Chatterjee et al., 2021). Interestingly, a 48 h pretreatment with the STEP inhibitor TC-2153 prior to Aβ treatment in cortical neurons significantly minimized the loss of dendritic complexity (Chatterjee et al., 2021). Moreover, in two studies using presynaptic and postsynaptic markers colocalization puncta to indicate the presence of synapse, it was also reported that Aβ-treated neurons showed significantly fewer colocalized synaptic puncta than control neurons (Kono et al., 2019), and that pretreatment with TC-2153 significantly increased the colocalized synaptic puncta in Aβ-treated neurons, and rescued the loss of dendritic spine density in 3×Tg-AD mice (Chatterjee et al., 2021).

Aβ and STEP also influence dendritic spines via their effect on actin. It was recently reported that upon Aβ exposure, fibrillar actin (F-actin), a major cytoskeletal protein that determines the shape of spines, depolymerizes to globular actin (G-actin) and, therefore, contributes to the decrease and collapse of spines (Kommaddi et al., 2018). Interestingly, the phosphorylation of the STEP substrate, SPIN90, leads to its binding with cofilin thereby reducing the actin-depolymerizing activity of cofilin (Cho et al., 2013a). However, when SPIN90 is dephosphorylated by STEP, it dissociates from cofilin leading to cofilin activation and thus, actin depolymerization, therefore, contributing to spine collapse (Figures 3, 4; Cho et al., 2013b). Furthermore, it was also reported that phosphorylated SPIN90 interacts with scaffolding proteins PSD-95 and Shank in the post-synaptic compartment. There are also substantial evidences showing the role of PSD-95 in increasing spine density as well as the number of synapses (El-Husseini et al., 2000), and of Shank in promoting spine maturation and enlargement (Sala et al., 2001). Consistent with these findings, a study reported a loss of synaptic clustering with both Shank and PSD-95 following SPIN90 dephosphorylation by STEP, that led to downregulation of dendritic spines in terms of size and density (Cho et al., 2013a). It should also be noted that inhibition of STEP with TC-2153 was able to improve dendritic spine abnormalities in *Fmr1* KO cultures and spine density in *Fmr1* KO mice model of FXS (Chatterjee et al., 2018).

Together, the data reviewed here strongly suggest that STEP is centrally involved in the loss of dendritic complexity and decrease spine density observed in AD patients as well as AD animal models and even in other neurological disorders.

## Step in Synaptic Plasticity and Cognitive Impairment

Cognitive and behavioral impairments are some of the primary and main clinical manifestations of neurodegenerative disorders like AD. Synaptic dysfunctions seen as synapse loss seem to significantly correlate with the functional and cognitive deficits observed in different stages of AD (Terry et al., 1991). Numerous studies have highlighted the role of STEP in the alterations of cognitive function in AD. For example, the Tg-2576 AD model mouse line carries mutations in APP that are found in early onset familial AD (Mirra et al., 1991; Terry et al., 1991; Khachaturian, 2006). These mice show normal cognitive functions at 3 months of age, however, at 10 months of age they exhibit significant cognitive impairments (Hsiao et al., 1996). Interestingly, the levels of STEP were found to be normal in these mice at 3 months but significantly elevated at 10 moths (Kurup et al., 2010), suggesting an implication of STEP in the observed alterations in these animals. Similarly, in 3×Tg-AD mouse model that has the same mutation with the Tg-2576 in addition to the presenilin and tau mutations, it was also found that the levels of STEP were normal at early stage of life, but increased at late stage and this change went together with the appearance of behavioral alterations. This has been corroborated by reports which showed that in 3×Tg-AD mice, STEP activity is significantly elevated after 6 months of age, which coincides with the start of memory deficits (Zhang et al., 2010). Moreover, crossing STEP KO mice with 3×Tg-AD mice prevents these cognitive alterations (Kurup et al., 2010; Zhang et al., 2010). These STEP KO mice have enhanced learning abilities including hippocampal-dependent learning (Venkitaramani et al., 2011) as well as amygdala-dependent learning (Olausson et al., 2012), indicating that elevated levels of STEP might disrupt synaptic plasticity and thus learning and memory formation. Interestingly, the STEP inhibitor, TC-2153, was reported to significantly rescue cognitive impairments (Xu et al., 2014) and the loss of dendritic spine density in 3×Tg-AD (Chatterjee et al., 2021), suggesting that inhibition of STEP might at least decrease the progression of neuronal deterioration in these AD mice models.

From the data reviewed above, it is clear that the loss of STEP leads to increased phosphorylation of its substrates including NMDA and AMPA receptors as well as ERK1/2, Fyn and Pyk2. Thus, it is logical to stipulate that loss of STEP could favor learning and memory. In line with this idea, a study reported that in a water maze reversal training task, STEP KO mice showed significantly better performance than WT (Venkitaramani et al., 2011), suggesting a higher degree of cognitive flexibility in STEP KO mice. The same study also revealed that in the water-escape motivated radial arm maze, STEP KO mice also outperformed WT mice. This test simultaneously evaluates spatial working and reference memories, and during the first 2 days of training in this test, STEP KO mice committed fewer reference and working memory errors compared with WT mice (Venkitaramani et al., 2011). Moreover, fear conditioning that tests amygdala-dependent memory showed that STEP KO mice exhibited a greater degree of fear memory (Olausson et al., 2012). However, no significant differences were found between these two groups when evaluating anxiety, motor coordination, and motor learning (Venkitaramani et al., 2011). This is in line with other studies showing that animals with increased expression or activation of STEP substrates like GluN2B, GluA1 and ERK1/2 had enhanced memory in MWM, fear conditioning and novel object recognition tasks (Tang et al., 1999; Wang et al., 2004; Okun et al., 2010). Pyk2 lies upstream of Fyn and when Pyk2 is activated following its phosphorylation at Tyr402, it phosphorylates and activates Fyn, which can then phosphorylate GluN2B at Tyr1472. Consequently, activation of Pyk2 leads to a greater phosphorylation and increased surface expression of GluN2B (Le et al., 2006), as well as enhanced phosphorylation of ERK1/2 (Nicodemo et al., 2010). Interestingly, the inhibition of Pyk2 results in blockage of LTP induction (Huang et al., 2001). As discussed earlier, Pyk2 is a substrate of STEP, and thus Pyk2 dephosphorylation by STEP would oppose these processes and impairs synaptic plasticity. In support of this, upregulated phosphorylation of Pyk2 was reported in STEP KO mice (Venkitaramani et al., 2011).

Numerous studies have suggested that BDNF and its receptor TrkB signaling alterations were also implicated in synaptic plasticity and memory impairments, and evidence suggested that this might be related to STEP dysregulation. For example, it was reported that the decreased BDNF level in AD was associated with reduced cortical cholinergic synapses, emphasizing the fact that dysregulation of BDNF might affect cholinergic synapses and thus synaptic plasticity (Amidfar et al., 2020). Recently, it was shown that upregulation of BDNF/TrkB mRNAs expression in the hippocampus is associated with improvement of memory (Amidfar et al., 2018). Moreover, alterations in BDNF expression and BDNF/TrkB signaling pathway might induce synapse loss and the consequent cognitive dysfunction (Song et al., 2015), while early downregulation of BDNF in AD was associated with the severity of cognitive impairments (Peng et al., 2005; Garzon and Fahnestock, 2007). In addition, BDNF/TrkB deprivation was recently found to activate JAK2/STAT3 pathway, leading to the upregulation of C/EBPβ, which in turn, increased the expression of asparaginyl endopeptidase (AEP), resulting in the cleavage of both APP and Tau, thus aggravating neuronal loss. Interestingly, inhibition of this cascade was able to rescue synaptic plasticity and cognitive impairments (Wang et al., 2019). CREB was shown to induce transcription and translation of BDNF (which binds to TrKB) leading to the phosphorylation of AMPA and NMDA receptors, while Aβ-induced inactivation of PKA/Akt inactivates CREB, and induces deficient BDNF/TrKB signaling leading to hippocampal synapse loss, synaptic plasticity and memory impairments in AD (Amidfar et al., 2020). Interestingly, it was found that BDNF/TrkB signaling can induce a decrease in the protein level of STEP in primary cortical neurons, via rapid ubiquitination and degradation of STEP, while downregulation of BDNF in cell and animal models increased the level of STEP (Saavedra et al., 2016; Xu et al., 2016). Moreover, the use of TrkB antagonist led to STEP accumulation and impaired long-term memory formation (Saavedra et al., 2019). The levels of Tyr phosphorylation of GluN2B and pERK1/2 were also increased in neuronal cultures following BDNF treatment or TrkB activation (Saavedra et al., 2016; Xu et al., 2016). Together, these studies indicate that the increased STEP in AD patients and animal models might possibly reflect the alterations in BDNF/TrkB signaling. In support of this hypothesis, it was reported that the TrkB signaling activator 7,8-DHF as well as the STEP inhibitor TC-2153 both ameliorated motor hyperactivity and Tyr phosphorylation of STEP substrates in BDNF^±^ mice (Xu et al., 2016).

Moreover, in other neurological disorder models, either pharmacological or genetic inhibition of STEP was able to ameliorate behavioral alterations. For example, it was found that inhibition of STEP improved locomotion, hyperactivity, memory, novel object recognition, anxiety and sociability observed in different models of SZ as well as in a model of FXS (Chatterjee et al., 2018; Xu et al., 2018). Furthermore, genetic deletion of STEP could delay the onset of motor dysfunction and prevent the appearance of cognitive deficits in R6/1 mice of HD, and this effect was associated with an increase in pERK1/2 levels and a reduction in the size of mHTT aggregates, in both the striatum and CA1 hippocampal region. Moreover, pharmacological inhibition of STEP with TC-2153 improved cognitive function in these mice (García-Forn et al., 2018).

## Step Alterations in AD and Other Neurological Diseases

STriatal-Enriched protein tyrosine Phosphatase is highly expressed in different brain regions except in the cerebellum, thus, it is not surprising that many studies have evaluated and confirmed the implications of STEP in several neurodegenerative disorders (Table 2), including AD (Kurup et al., 2010; Zhang et al., 2010), Parkinson’s disease (Kurup et al., 2015), Huntington’s disease (Gladding et al., 2012), schizophrenia (Xu et al., 2018), fragile X syndrome (Chatterjee et al., 2018), age-related memory decline (Castonguay et al., 2018), depressive disorders (Elizabeth and Alexander, 2017) and in mouse model of Sepsis-Associated Encephalopathy (Zong et al., 2019).

**TABLE 2:** STEP alteration mechanisms in neurological disorders and the therapeutic strategies.

**Disease**	**Model (s)**	**Changes in STEP**	**Mechanism of STEP alterations**	**Intervention strategies and outcome**	**References**
Alzheimer’s disease	Humans, Mice & Cells	↑	Aβ-induced dysregulation of UPS, activation of α7nAChRs and dysregulation of BDNF/TrkB signaling	Pharmacologic (TC-2153) or genetic inhibition (KO, KD) of STEP improved phosphorylation of GluN2B, GluA2, ERK1/2, Fyn, Pyk2, synaptic connectivity, BDNF and cognitive functions	Dineley et al., 2001; Stevens et al., 2003; Lacor et al., 2004; Chin et al., 2005; Almeida et al., 2006; Venkitaramani et al., 2007, 2011; Tseng et al., 2008; Kurup et al., 2010; Zhang et al., 2010, 2013; Olausson et al., 2012; Xu et al., 2014, 2016; Saavedra et al., 2016
Parkinson’s disease	Humans, Rats, Mice & Cells	↑	Disrupted UPS associated with mutation/decreased activity of parkin, a product of PARK2 gene and downregulation of BDNF/TrkB signaling	Activation of BDNF signling decreases STEP level and activity which results in upregulation of pERK, pCREB and BDNF	Kitada et al., 2009; Chagniel et al., 2014; Kurup et al., 2015; Saavedra et al., 2016; Xu et al., 2016
Huntington’s disease	Mice	↑↓	Downregulation: enhanced PKA and reduced PP2B activities; Upregulation: decrease DARPP-32 levels	Genetic deletion of STEP delayed onset of motor and cognitive symptoms. TC-2153 improved cognition, TAT-STEP increased VGLUT1-GluN2B colocalization, Y1472GluN2B and BDNF expression	Desplats et al., 2006; Hodges et al., 2006; Saavedra et al., 2011; Gladding et al., 2012; García-Forn et al., 2018
Schizophrenia	Humans, Mice & Cells	↑	NRG1 mutation and NRG1/ErbB4 signaling abnormality	Neuroleptic drugs or genetic inhibition of STEP improved synaptic proteins (NRG1, GluN2B, Pyk2 and ERK1/2) and behavioral deficits	Stefansson et al., 2004; Barros et al., 2009; Belforte et al., 2010; Carty et al., 2012; Goebel-Goody et al., 2012a; Loh et al., 2013; Xu et al., 2018
Fragile X syndrome	Mice & Cells	↑	Defect in the *Fmr1* gene causes deficiency of FMRP, the protein product of *Fmr1*, which normally binds to and suppresses mRNAs translation including that of STEP; mGlu signaling alteration	TC-2153 or genetic deletion of Fmr1 or STEP/Fmr1 double KO improved exaggerated LTD, audiogenic seizure incidences, c-Fos-positive neurons hyperactivity, anxiety and synaptic aberrations	Huber et al., 2002; Hou et al., 2006; Gross et al., 2010; Darnell et al., 2011; Goebel-Goody et al., 2012b; Chatterjee et al., 2018
Age-related memory decline	Humans, Monkeys, Mice & Cells	↑	Abnormalities in the UPS as well as alteration in the NMDAR and ERK signaling pathways	Up- or down-regulation, led to worsening or alleviation of age-related memory deficits, respectively; TC-2153 improved synaptic proteins and alleviated cognitive impairments	Castonguay et al., 2018
Depressive disorders	Humans & Mouse	↑*	Downregulation of BDNF signaling pathway	TC-2153 mitigates depressive-like symptoms in mice via decreasing 5-HT_2__A_ receptor and increasing BDNF	Kulikov et al., 2012; Elizabeth and Alexander, 2017; Kulikova et al., 2018

*There is a controversy regarding the level of STEP in depressive disorders with elevation of this enzyme seen in mice but no changes were observed in postmortem human study. Therefore, further researches are needed to draw a conclusion.

### Alzheimer’s Disease

There is accumulating evidence that STEP activity, Aβ levels, and synapse regulation are closely related. The level of STEP has been reported to be elevated in AD, the most common neurodegenerative disorder, including in the brain of post-mortem AD patients and in several AD mice models like the Tg2576 (Kurup et al., 2010), J20 (Chin et al., 2005), APP/PS1 (Zhang et al., 2013), and 3×Tg-AD mice (Zhang et al., 2010). The increment in STEP is believed to be the consequence of increased levels of Aβ in AD, which leads to the dysregulation of the UPS and activation of α7nAChRs, both of which eventually lead to an increase in the expression levels and activity of STEP (Kurup et al., 2010; Zhang et al., 2013). Aβ can bind to and activate α7nAChRs (Dineley et al., 2001; Stevens et al., 2003; Lacor et al., 2004), triggering calcium influx that activates PP2B, that in turn, inactivates DARPP-32, leading to the activation of PP1, which then dephosphorylates and activates STEP (Figure 4; Snyder et al., 2005). Consequently, the activated STEP can readily bind to and dephosphorylate its target proteins. It has been shown that transgenic AD mouse models as well as neurons exposed to Aβ show loss of spines (Lacor et al., 2004; Calabrese et al., 2007; Shankar et al., 2007; Wei et al., 2010; Spires-Jones and Hyman, 2014). Consistently, neurons treated with Aβ or those that overexpress APP exhibit decreased glutamatergic transmission (Ting et al., 2007). Moreover, exogenous Aβ treatment was shown to induce endocytosis of NMDA receptors through a STEP-dependent pathway (Kurup et al., 2010). Additionally, NMDA receptors are involved in the regulation of dendritic spine density and morphology (Ultanir et al., 2007), suggesting that STEP-mediated downregulation of NMDA receptors may contribute to the loss of synaptic density in AD. It was also believed that Aβ oligomers might also cause synaptic dysfunction by inducing PP2B-dependent internalization of AMPA receptor (Hsieh et al., 2006). Moreover, it was recently reported that STEP binding results in the dephosphorylation and decreased synaptic expression of GluA2, while synaptic expression of GluA2 is increased in the brain of STEP-KO mice (Won et al., 2019). This could therefore be at least one of the ways Aβ mediates AMPA receptor internalization and synaptic impairment. It was also observed that both neuronal cultures treated with Aβ and AD mouse models have an accumulation of active STEP (Chin et al., 2005; Kurup et al., 2010; Zhang et al., 2010, 2013) associated with the Aβ-mediated impairment of the UPS (Almeida et al., 2006; Tseng et al., 2008), since neither transcription nor translation of STEP was altered. Moreover, studies have reported an increase in STEP level as a result of Aβ-mediated disruption of the UPS pathway (Venkitaramani et al., 2007; Kurup et al., 2010). Thus, in AD, a decrease in its degradation together with an increase in its dephosphorylation would be in part responsible for the significant increase in the level of active STEP. The net outcome of increased active STEP in the brain is the dephosphorylation of GluN2B Tyr1472 and internalization of GluN1/GluN2B receptor complex (Snyder et al., 2005; Kurup et al., 2010; Zhang et al., 2010), dephosphorylation of the GluA2 subunit of AMPA receptor and internalization of GluA1/GluA2 complex (Zhang et al., 2008; Won et al., 2019), dephosphorylation and inactivation of Fyn, Pyk2 (Nguyen et al., 2002; Xu et al., 2012), ERK1/2 (Paul et al., 2003; Valjent et al., 2005; Paul and Connor, 2010), SPIN90 (Cho et al., 2013b; Figure 4). Taken together, these indicate that STEP mediates the Aβ induced synaptic plasticity and cognitive impairments seen in AD animal models as well as AD patients via inactivation of synapse related proteins and endocytosis of both NMDA and AMPA receptors.

As summarized above, substantial evidence has highlighted the implication of BDNF in AD (Peng et al., 2005; Garzon and Fahnestock, 2007; Song et al., 2015; Amidfar et al., 2018; Wang et al., 2019). Both protein levels and mRNA expression of BDNF were reported to be reduced in postmortem brain samples of AD patients (Tanila, 2017) and in animal models of AD resulting in decreased cholinergic synapses (Iulita et al., 2017; Amidfar et al., 2020). It is known that CREB could induce the transcription and translation of BDNF leading to the phosphorylation of AMPA and NMDA receptors, while Aβ can inactivate the PKA and dephosphorylate Akt which would inactivate CREB and induce deficit in BDNF pathway leading to hippocampal synaptic loss, synaptic plasticity impairment and memory deficit in AD (Amidfar et al., 2020). Activation of BDNF/TrkB signaling can induce a decrease in the protein level of STEP and increased phosphorylation of its substrates, while downregulation of BDNF had the opposite effect (Saavedra et al., 2016; Xu et al., 2016). The level of STEP was reported to be increased while that of BDNF to be decreased in AD patients and animal models. This is an indication that the decreased BDNF and/or BDNF/TrkB signaling in AD could possibly be, at least in part, responsible for the increase in the STEP level, suggesting that, in AD, Aβ induces alterations in BDNF/TrkB signaling to alter synaptic morphology possibly via increasing activation and protein level of STEP.

Another way that STEP mediates the Aβ-induced cognitive impairment is via the STEP dephosphorylation of SPIN90 that induce F- to G-actin depolymerization of cofilin (Cho et al., 2013a, b; Kommaddi et al., 2018). Moreover, STEP also disrupts the interaction of PSD-95 and Shank that is important in the maintenance of dendritic spine integrity (El-Husseini et al., 2000; Sala et al., 2001). These together, lead to decreased size and density of dendritic spines and eventually spine collapse. This evidence highlights an additional pathway via which Aβ/STEP triggers AD pathology via reduced dendritic spine density and synapse loss which appears as the learning and memory and cognitive impairments seen in AD patients and AD animal models.

### Parkinson’s Disease

Studies have also reported an upregulation of STEP in PD brain as well as in MPTP-induced PD model (Kurup et al., 2015), the next most common neurodegenerative disorder after AD, which is characterized by loss of dopaminergic neurons in the substantia nigra and dopamine depletion in the striatum (Saiki et al., 2012). The increase in STEP is correlated with a decrease in the phosphorylation of ERK1/2 and CREB, an effect that might contribute to the synaptic and cognitive impairments seen in PD (Kurup et al., 2015). There is substantial evidence indicating that a decrease in the expression of parkin, a product of *PARK2* gene, is involved in the genetic forms of PD. This is supported by the fact that mutations of the *PARK2* gene result in an autosomal recessive juvenile parkinsonism with early onset of PD symptoms (Shimura et al., 2000; Tanaka et al., 2004), and alterations in the activity of parkin were involved in both familial and sporadic PD (Sriram et al., 2005; Dawson and Dawson, 2010, 2014). Moreover, the dopaminergic neurotoxins MPP^+^ and MPTP induced alterations in the levels or activity of parkin with consequent accumulation in pathogenic parkin substrates such as AIMP2 and PARIS (Ko et al., 2010; Imam et al., 2011; Dawson and Dawson, 2014). Interestingly, the level of STEP is increased in both human PD samples and PD models (Kurup et al., 2015). STEP is normally degraded via the ubiquitin proteasome system, and it was found that parkin is an E3 ligase that ubiquitinates STEP *in vivo* and *in vitro*, suggesting that the decrease in parkin activity might be responsible for the observed increase in STEP protein in PD (Kurup et al., 2015). In support of this, it was found that shRNA-downregulated and parkin KO rats showed an increase in the level of STEP, and that STEP upregulation was associated with down-regulation of synaptic proteins in the striatum (Kurup et al., 2015). Interestingly, the striatum of *PARK2* KO mice showed a decrease in evoked dopamine release and resulted in impaired LTP and LTD in striatal medium spiny neurons (Kitada et al., 2009). As reviewed above, increased STEP can impair LTP and LTD via inactivation of its substrates including GluN2B, GluA2, ERK1/2, Fyn and others. Thus, these indicate that the reduction in dopamine release in *PARK2* KO mice might decrease the PKA-induced STEP phosphorylation (inactivation), resulting in increased STEP activity and consequent cognitive deficits seen in PD. Consistent with this, it was found that PKA-mediated phosphorylation of STEP correlated with enhanced motor learning, and attenuating striatal STEP activity via PKA phosphorylation was believed to be associated with a striatal molecular pathway involved in the consolidation of complex motor skills during motor learning (Chagniel et al., 2014).

Moreover, it was also found that BDNF signaling could lead to a rapid ubiquitination and degradation of STEP via binding to its receptor TrkB, which results in the activation of the phospholipase Cγ and protein kinase C (PKC) pathways (Saavedra et al., 2016; Xu et al., 2016). Decreased neurotrophic factor signaling has been proposed to be implicated in the pathophysiology of PD (Baquet et al., 2005; Rangasamy et al., 2010; He et al., 2013), and STEP levels are increased in human PD samples and MPTP-lesioned mice (Kurup et al., 2015). These are indications that the increase in STEP expression levels in PD could be the result of decreased neurotrophic factor signaling which may probably contribute to PD pathophysiology. In line with this, previous studies reported that inhibiting PTPs protected dopaminergic neurons from PD toxins by activating ERK1/2 via increasing BDNF signaling (Lu et al., 2002), and that phosphorylation of ERK1/2 and CREB was decreased in sporadic PD samples (Kurup et al., 2015). In summary, BDNF leads to a downregulation of the protein level of STEP, whereas increased STEP levels result in decreased pERK1/2/pCREB-mediated expression of BDNF, suggesting a feedback regulation.

### Huntington’s Disease

STriatal-Enriched protein tyrosine Phosphatase alterations have also been documented in Huntington’s Disease (HD), a genetic disorder characterized by progressive neurodegeneration, poor muscle coordination, mood disorders, and dementia (Ross and Tabrizi, 2011). There are controversies in the dysregulation of STEP in HD as both downregulation and upregulation have been observed. For instance, a study by Saavedra et al., reported a decreased STEP activity. In their study, these authors showed that with age a decrease in protein level of STEP was observed in the striatum and cortex of R6/1 HD mouse model, while increased STEP phosphorylation was seen in striatum, cortex and hippocampus (Saavedra et al., 2011). These changes together resulted in decreased STEP activity which correlated with enhanced PKA and reduced PP2B activities as well as an increased phosphorylation of two STEP substrates ERK1/2 and p38. Downregulation of STEP activity was also reported in other HD mouse models including R6/2, Tet/HD^94^, and Hdh^*Q*7/Q111^ (Saavedra et al., 2011). Interestingly, it was reported that R6/1 mice showed resistance to quinpirole, an NMDA receptor agonist, induced excitotoxicity (Hansson et al., 2001), while co-administration of quinpirole with WT TAT-STEP exacerbated excitotoxicity in both WT and R6/1 mice (Saavedra et al., 2011). These findings suggest that STEP increases the vulnerability of striatal neurons to excitotoxity and that the decreased STEP in HD mouse models may confer to these mice their resistance to excitotoxicity. In support of this, a decrease in the mRNA levels of STEP was previously reported in the caudate nucleus and cortex of HD patients (Hodges et al., 2006) as well as in the striatum of R6/1 mice (Desplats et al., 2006).

On the other hand, a recent study revealed that genetic deletion of STEP delayed both the onset of motor dysfunction and the decrease of striatal DARPP-32 levels, and prevented the appearance of cognitive deficits in R6/1 mice (García-Forn et al., 2018). Importantly, this was associated with an increase in pERK1/2 levels and a reduction in the size of mHTT aggregates, in both the striatum and CA1 hippocampal region. Moreover, pharmacological inhibition of STEP with TC-2153 improved cognitive function in these mice (García-Forn et al., 2018). In addition, another study also reported a significantly increased synaptic STEP activity in the striatum of YAC128, a mouse model of HD, compared to WT mice, and this correlated with decreased GluN2B Y1472 phosphorylation (Gladding et al., 2012). Moreover, calpain activation leads to GluN2B cleavage at both synaptic and extra-synaptic sites, thereby further decreasing surface expression of GluN2B. These authors also showed that in striatal neuron cultures C-S mutant TAT-STEP (non-active STEP) significantly increased VGLUT1-GluN2B colocalization, as well as increasing Try1472 phosphorylation and synaptic GluN2B expression, while *in vivo* STEP inhibition also increased synaptic GluN2B expression in the YAC128 striatum (Gladding et al., 2012). Of interest is the fact that combined inhibition of STEP and calpain reduced extra-synaptic, but increases synaptic expression of GluN2B in the YAC128 striatum (Gladding et al., 2012). These results together suggest that upregulated activity of both STEP and calpain could be responsible for the mis-localization of NMDAR from synaptic to extra-synaptic site in YAC128 mouse model of HD.

The discrepancies reported in the dysregulation of the level of STEP in these HD animal models could be attributed to the differential mechanisms involved. Whereas Saavedra et al., focused on the dopaminergic activation of PKA, García-Forn et al., highlighted the role of DARPP-32 pathway. However, further investigations are needed to clarify the existing differences. Another potential reason could be the HD animal model used.

### Fragile-X Syndrome

Fragile-X syndrome (FXS) is another neurological disorder, the leading cause of inherited intellectual disability, with core symptoms including cognitive deficits, anxiety and seizures. This condition is mainly due to a genetic alteration that suppresses the transcription of *Fmr1* gene. Interestingly, this gene was shown to be related to learning and memory functions as suggested by the fact that *Fmr1* KO mice have decreased surface expression of NMDA and AMPA receptors (Suvrathan et al., 2010; Eadie et al., 2012). FMRP, the product of *Fmr1* expression, normally binds to and suppresses dendritic translation of a myriad of mRNAs following mGlu5 stimulation (Antar and Bassell, 2003; Bear et al., 2004). Therefore, due to the absence of FMRP in FXS, the translation of many of these mRNAs is upregulated including STEP mRNA (Huber et al., 2002; Hou et al., 2006; Gross et al., 2010; Darnell et al., 2011; Chatterjee et al., 2018). It was found that STEP mRNA can associate with FMRP (Darnell et al., 2011; Goebel-Goody et al., 2012a), and upregulation of STEP translation was demonstrated in *Fmr1* KO (Goebel-Goody et al., 2012b; Chatterjee et al., 2018). Moreover, the mGlu5 agonist, DHPG, leads to a rapid and dose-dependent increase in STEP translation (Zhang et al., 2008), while STEP inhibition was found to be beneficial in maintaining synaptic homeostasis in the hippocampal neurons in mouse models of FXS (Chatterjee et al., 2018). Interestingly, a decrease in both audiogenic seizures and seizure-induced c-Fos-positive neurons in the periaqueductal gray matter were observed in STEP/*Fmr1* double KO mice compared to *Fmr1* KO (Goebel-Goody et al., 2012b). In addition, the STEP inhibitor TC-2153 was found to reverse audiogenic seizure incidences, hyperactivity, mGlu5-mediated exaggerated LTD, ameliorated behavioral alterations like anxiety, and sociability in *Fmr1* KO mice, as well as improved synaptic aberrations both *in vivo* and in *Fmr1* KO neuronal cultures (Chatterjee et al., 2018). These results imply that the translation of STEP is increased in FXS model as a result of *Fmr1* gene downregulation, and decreased STEP expression is associated with improvement of the cognitive impairments.

### Schizophrenia

Alterations in STEP are also observed in schizophrenia (SZ), a neurological disease with complex etiology, where neuronal dysfunction, genetics, and environment come together (Tsuang et al., 2001; van de Leemput et al., 2016). Cognitive deficits are some of the symptoms of patients with SZ, and one proposed mechanism involved in the behavioral alterations in SZ is decreased NMDAR function and/or decreased surface expression of NMDARs (Xu et al., 2018). Several studies have provided results that are consistent with this hypothesis. For instance, a study reported abnormalities in glutamate receptor density in postmortem SZ brains in the prefrontal cortex, temporal lobe, and thalamus (Goebel-Goody et al., 2012a). In addition, a decrease in the mRNA level of GluN1 was reported in postmortem SZ brain, and it was correlated with antemortem severity of cognitive impairment (Humphries et al., 1996; Catts et al., 2016), while d-serine and glycine (facilitators of NMDRs activation) administration improved symptoms in medicated SZ patients (Kantrowitz et al., 2010; Balu and Coyle, 2015). Moreover, SZ-like behaviors were reported in mice with reduced expression of NMDAR (Belforte et al., 2010), and in persons taking non-competitive NMDAR antagonists like phencyclidine (PCP) or ketamine (Goebel-Goody et al., 2012a). In line with this, it was found that NRG1, a growth factor that promotes phosphorylation and surface retention of NMDARs was mutated in patients with SZ (Stefansson et al., 2004; Loh et al., 2013). Upon binding to its receptor (ErbB4), NRG1 normally activates Fyn leading to Tyr1472 phosphorylation and surface expression of GluN2B (Bjarnadottir et al., 2007). This finding is supported by a study where NRG1/ErbB4 signaling was revealed to promote synaptic incorporation of NMDARs via NRG1/ErbB4-stimulated binding of PSD-95 to Erbin (Barros et al., 2009). Moreover, Tyr1472 phosphorylation of GluN2B was found to be decreased in NRG1 heterozygous mice (Bjarnadottir et al., 2007; Xu et al., 2018). Also, the locomotor and cognitive deficits induced by the NMDA receptor antagonist MK801 and PCP were attenuated in STEP KO mice (Carty et al., 2012). All these suggest that increase in STEP might be responsible for the SZ symptoms via decreasing surface NMDA receptors. However, an opposite result was reported by Pitcher et al., where stimulation of NRG1 was reported to attenuate NMDAR activity via suppressing Src-mediated phosphorylation of GluN2B and potentiation of NMDARs (Pitcher et al., 2011). This evidence, therefore, suggests that NRG1 hyperactivation in SZ leads to NMDAR hypofunction (Hahn et al., 2006). The above-mentioned discrepancy could be attributed to differential effect of the two different kinases Fyn and Src evaluated in these two studies, even though these are both SFKs, and thus further investigation is needed to explore the pathways involved. It is clear that NMDA receptors’ hypofunction is implicated in SZ, and STEP can decrease both surface expression and activity of these receptors. Thus, STEP might also be involved in SZ. Interestingly, it was recently found that the protein levels of STEP are significantly upregulated in heterozygous NRG1 and CNS-specific ErbB2/4 KO mouse models of SZ, hiPSC neurons from forebrain of SZ patients (Xu et al., 2018), and in the cortex of postmortem SZ patients (Carty et al., 2012). Also, genetically eliminating STEP in mice decreased their susceptibility to PCP-induced locomotor activity and cognitive deficits when compared with WT mice (Carty et al., 2012; Xu et al., 2018). Moreover, treatment of WT mice with neuroleptics (haloperidol, clozapine, and risperidone), used to treat SZ, increased the PKA-mediated Ser221 phosphorylation of STEP via DARPP-32/PP1 pathway, along with increased phosphorylation of GluN2B, Pyk2, and ERK1/2, and surface expression of GluN2B (Carty et al., 2012). Additionally, both pharmacological and genetic inhibition of STEP increased the phosphorylation of NRG1, GluN2B and ERK1/2 (Xu et al., 2018). Together, the data summarized here clearly indicate the implication of STEP in the etiopathogenesis of SZ.

## Step as Therapeutic Target

Several studies have documented the involvement of STEP in several neuropsychiatric disorders, including Alzheimer’s disease (Kurup et al., 2010; Zhang et al., 2010), Parkinson’s disease (Kurup et al., 2015), Huntington’s disease (Gladding et al., 2012), Schizophrenia (Xu et al., 2018), Fragile X syndrome (Chatterjee et al., 2018), age-related memory decline (Castonguay et al., 2018) and depressive disorders (Elizabeth and Alexander, 2017; Table 2). Most of these disorders have reported an increase in the STEP protein level and/or activity, thus making STEP an important therapeutic target leading to the search and development of STEP inhibitors. The level of STEP has been reported to be elevated in AD, including in post-mortem AD patients and several AD mice models (Chin et al., 2005; Kurup et al., 2010; Zhang et al., 2010, 2013). This increase in STEP is believed to be the consequence of the accumulation of Aβ that leads to the dysregulation of the UPS and activation of α7nAChRs, both of which eventually lead to increased levels of activated STEP (Kurup et al., 2010; Zhang et al., 2013). Interestingly, it was found that pharmacological (TC-2153) as well as genetic inhibition of STEP were able to ameliorate cognitive function and hippocampal memory in the 3×Tg-AD mouse model (Zhang et al., 2010; Xu et al., 2014). Moreover, it was reported that STEP inhibition not only improved cognitive functions, but also increased synaptic connectivity in both cell cultures and 3×Tg-AD mouse model (Chatterjee et al., 2021). Similar results were also reported in SZ models, where inhibition of STEP was sufficient to improve both biochemical and behavioral deficits in these SZ mice models (Xu et al., 2018). Also, in mice model of FXS, pharmacological inhibition of STEP with TC-2153 was able to reverse mGlu5-mediated exaggerated LTD, audiogenic seizure incidences, hyperactivity, ameliorated behavioral alterations like anxiety and sociability in *Fmr1* KO mice, as well as improved synaptic aberrations both *in vivo* and in *Fmr1* KO neuronal cultures (Chatterjee et al., 2018). TC-2153 was also reported to have antidepressant-like effect via decreasing both activity and protein level of the serotoninergic 5-HT_2__A_ receptor in the hippocampus and frontal cortex, but not in the striatum (Kulikova et al., 2018), and increasing BNDF in brains of mice genetically predisposed to depressive-like behavior (Kulikov et al., 2012). As reported earlier, BDNF was found to induce rapid STEP ubiquitination pathways (Saavedra et al., 2016; Xu et al., 2016). Therefore, the inhibition of STEP might be responsible for the increase in BDNF, which can further induce rapid degradation of STEP resulting in decreased STEP activity, and might consequently increase the phosphorylation of STEP substrates. In line with this, it was revealed that treatment of cortical neurons with TC-2153 as well as TC-2153 injection of mice induced a significant improvement in the Tyr phosphorylation of STEP substrates GluN2B, Pyk2, and ERK1/2, and behavioral deficits (Xu et al., 2014). In addition, crossing STEP KO mice with 3×Tg-AD mice improved cognitive alterations (Kurup et al., 2010; Zhang et al., 2010), and the STEP KO mice have facilitated learning abilities in hippocampal-dependent (Venkitaramani et al., 2011) as well as amygdala-dependent learning (Olausson et al., 2012). Overall, these findings indicate that STEP regulates learning and memory signaling pathways, and that elevated levels of STEP might disrupt synaptic plasticity and thus learning and memory formation.

TC-2153 is the currently most experimentally used STEP inhibitor. Interestingly, phosphatase assay following TC-2153 treatment showed selectivity toward STEP inhibition among an array of PTPs, including He-PTP and PTP-SL (Xu et al., 2014). Moreover, following injection with TC-2153, WT and STEP KO mice showed a significant increase in Tyr phosphorylation of ERK1/2 and Pyk2, only in the frontal cortex and hippocampus, but not in tissues outside of the brain or in the cerebellum which lack STEP (Xu et al., 2014). These together, indicate the selectivity of TC-2153 toward STEP, as compared to other phosphatases. The inhibitory effect of TC-2153 on STEP was speculated to probably be through the formation of a covalent bond within the catalytic domain of STEP involving a cysteine residue (Boivin et al., 2010). In line with this, mutation of the active cysteine to sulfur (C-S TAT-STEP) was found to produce an inactive form of STEP (Gladding et al., 2012).

Other STEP inhibitors are also under investigation and development. For instance, a substrate-based method called substrate activity screening (SAS) was also used to develop a low molecular weight STEP inhibitor 12t, which resulted in significant levels of STEP inhibition in rat cortical neurons (Baguley et al., 2013). The X-ray crystal structures of some of these STEP inhibitors were disclosed and these inhibitors showed a 15-60-fold selectivity toward STEP across a series of phosphatases (Witten et al., 2017). Using machine learning based computational models, another study also predicted two major compounds as candidate STEP inhibitors that could be potentially used for AD treatment (Jamal et al., 2015). Recently, a group of researchers combined molecular dynamics simulations and fragment-centric topographical mapping to identify transiently open cryptic pockets based on which they identified 12 new STEP inhibitors. Furthermore, they showed that the two most potent compounds ST2-5 and ST3-5 (analogs of ST2 and ST3) could reversibly bind and competitively inhibit STEP with selectivity for STEP against a panel of protein phosphatases. Moreover, these potent inhibitors were found to modulate the Tyr phosphorylation of both ERK1/2 and Pyk2, have relatively no toxicity in PC12 cell cultures, and also favor cell differentiation and migration functions (Hou et al., 2020). However, most of these STEP inhibitors are at their embryonic developmental level and further rigorous studies are needed to evaluate their therapeutic efficacy, selectivity, specificity and safety. It is unfortunate to note that some still lack enough drug availabilities for further development.

Although nearly all discussed diseases reported increase in STEP activity leading to STEP inhibitor development, a decreased activity of STEP has also been reported and STEP activator could also be beneficial. Recently, the discovery of the first small molecule allosteric activator of STEP (BI-0314) that binds to the phosphatase domain has been reported (Tautermann et al., 2019). BI-0314 is a hSTEP specific positive allosteric modulator which, due to the low conservation of the residues in the allosteric site, has been shown to be inactive when tested on two other related tyrosine phosphatases (Tautermann et al., 2019). However, further studies are warranted before *in vivo* testing of this molecule is carried out.

## Summary and Concluding Remarks

From the data reviewed in this study, it is evident that increased STEP as a result of alteration of UPS, increased translation, as well as decreased phosphorylation (PKA and PP1) has been implicated in many neuropsychiatric diseases. This increase in STEP generally leads to a decreased phosphorylation of the STEP substrates which are basically found to be synapse related proteins. They include, but are not limited to, GluN2B, GluA2, GluA3, ERK1/2, p38, Fyn, Pyk2 and SPIN90. These alterations lead to internalization of NMDA and AMPA receptors, collapse and loss of dendritic spines together culminating into synaptic plasticity and learning and memory impairments, which are expressed in human patients and animal models of these diseases as cognitive deficits. In light of these observations, numerous studies have provided evidence of cognitive improvements following STEP inhibition. These improvements might be the reflection of improvements of healthy synaptic markers and morphology, reinforcing the role of STEP in AD and other neuropsychiatric pathologies. The observations that STEP is implicated in many neurological disorders, and the fact that modulating STEP level and activity have some beneficial effects in these disorders, make STEP a potential therapeutic target and led to the development of STEP inhibitors. These inhibitors are at their early developmental stage and, therefore, there is a long way to go before their clinical application. Although STEP inhibition has been reported, to be beneficial in improving both biochemical and behavioral parameters, it should be noted that, a study had reported that both genetic deletion and pharmacological inhibition of STEP were associated with thermal hyperalgesia and mechanical allodynia, accompanying the increased GluN2B Tyr1472 and ERK1/2 Thr202/Tyr204 phosphorylation in the lumbar spinal cord (Azkona et al., 2016). Moreover, a protective role of increased STEP activity has been reported, whereby a transient NMDA receptors stimulation increases STEP activity and appears to limit the duration of activation of the excitotoxicity and death related p38 MAPK and thus improving neuronal survival (Poddar et al., 2010). These findings suggest that the side effects of STEP inhibitors and the degree to which STEP should be inhibited have to be meticulously considered.

It has been over three decades since STEP was discovered to be involved in neurological diseases, however, there is still a lot to explore in the molecular mechanisms of STEP. For example, it will be interesting to investigate the molecular mechanisms responsible for initiating STEP ubiquitination which will further lead to a strategy in reducing STEP function. Moreover, discovering more STEP substrates and upstream modulators will also pave more opportunities in STEP therapeutics. Also, it was recently found that the activity of STEP is modulated by adenosine A_2__A_ receptor (A_2__A_R) in neuronal cells. A_2__A_R is a G protein-coupled receptor which is widely expressed in the brain where it regulates important functions, such as motor behavior and cognition (Chen et al., 2014; Pinna et al., 2018), and plays a key role in cell survival and neurodegeneration (Cunha, 2016). Interestingly, A_2__A_R stimulation was found to result in PKA activation (Fresco et al., 2004; Borea et al., 2018), and was involved in cocaine-induced stimulation of STEP (Chiodi et al., 2014). Moreover, A_2__A_R is considered as a promising target in the treatment of neuropsychiatric disorders, and so is STEP (Zhang et al., 2010; Xu et al., 2014, 2018; García-Forn et al., 2018). Recently, it was found that STEP activity was increased in NSEA_2__A_ (a transgenic rats overexpressing A2_*ARs*_ in the CNS) compared to WT rats (Mallozzi et al., 2020). STEP was also reported to mediate the cocaine-induced synaptic transmission depression probably via reducing AMPA- and NMDA-mediated excitatory post-synaptic currents (Chiodi et al., 2014).

## Author Contributions

YM and XW wrote the manuscript. FH, KE, and FZ assisted with data analysis and interpretation and critically read the manuscript. All authors contributed to the article and approved the submitted version.

## Conflict of Interest

The authors declare that the research was conducted in the absence of any commercial or financial relationships that could be construed as a potential conflict of interest.
